# Prevalence and risk factors for zoonotic helminth infection among humans and animals - Jos, Nigeria, 2005-2009

**Published:** 2012-05-12

**Authors:** Pius Stephen Ekong, Raymond Juryit, Ndahi Mwapu Dika, Patrick Nguku, Monica Musenero

**Affiliations:** 1 Epidemiology Section, National Veterinary Research Institute, Vom, Nigeria; 2 Plateau State Ministry of Health, Jos, Nigeria; 3 Federal Department of Livestock and Pest Control Services, Abuja, Nigeria; 4 Nigerian Field Epidemiology and Laboratory Training Program, Abuja, Nigeria; 5 African Field Epidemiology Network, Kampala, Uganda

**Keywords:** Zoonosis, helminth, prevalence, risk factor, Nigeria

## Abstract

**Background:**

Zoonotic infections are among the most common on earth and are responsible for over 60% of human infectious diseases, some of which are caused by helminth parasites. Infection may result from ingestion of infective stage of worms with food, contaminated soil; skin penetration or direct animal contact. This study estimates the prevalence of zoonotic helminth infections (ZHI) among animals and humans in Jos and identifies associated risk factors

**Methods:**

We reviewed laboratory records from five hospitals, one veterinary clinic and meat inspection record at abattoir in Jos from 2005-2009. Prevalence was defined as the observed frequency of cases of zoonotic helminth in the sampled population within the study period. Odd ratio analysis was used to identify factors associated with ZHI.

**Results:**

Of 6689 humans tested, 524 (7.8%) were positive. Observed ZHI are: *Ascaris species* (4.5%), *Taeniasis-Cysticercosis* (1.5%), *Schistosoma species* (1.1%), *Strongyloidosis* (0.09%). Among animals, 3520 (18.1%) of 19508 tested/observed were positive; including *Fasciola species* (12.7%), *Taeniasis-Cysticercosis* (5.0%), *Strongyloidosis* (0.4%), *Ascaris species* (0.04%). The risk of infection was higher among humans aged 6-19 (OR: 3.2; 95% CI: 2.0-5.2) and 20-60 (OR: 2.3; 95% CI: 1.7-3.9). Peri-urban dwellers are at higher risk (OR: 1.5; 95% CI: 1.3-1.9); and so are farmers.

**Conclusion:**

The prevalence of zoonotic helminth infection is high among humans and animals in Jos. Risk of infection are higher among human age 6-60, peri-urban dwellers and farmers. This calls for the formulation of workable collaboration between human and veterinary medical disciplines for better control of zoonotic helminth infections.

## Background

Zoonotic infections can be defined as infections of animals that are naturally transmissible to humans. As such they are worldwide and often spread with humans through their companion and domestic animals [1]. Zoonotic infections are among the most common on earth and are responsible for > 60 per cent of all human infectious diseases. Some of the most important and well-known human zoonoses are caused by worm or helminth parasites, including species of nematodes (trichinellosis), cestodes (cysticercosis, echinococcosis) and trematodes (schistosomiasis) [2]. Others include intestinal capillariasis, anisakidosis, eosinophilic enteritis, oesophagostomiasis and gnathostomiasis [3]. Tissue parasites of humans are still prevalent in most regions of the world, and are also seen more frequently in developed countries due to increasing travel patterns [4]. Echinococcus infections still account for hepatic and pulmonary pathology, cysticercosis is a major cause of seizures and epilepsy, and fascioliasis also causes significant liver pathology.

Human infection with these zoonotic helminths may result from ingesting food. This food may be meat containing the parasite (taeniasis; trichinosis); fish (diphyllobothriasis; *Diplogonorus granidis*; clonorchiasis; anisakiasis); invertebrates (paragonimiasis; angiostrogyliasis) or ingestion of the infective stage of the worm with contaminated soil (toxocariasis; hydatid) water or salad plants (fascioliasis; fasciolopsiasis; hydatid; toxocariasis); skin contact with contaminated soil / water containing active infective larvae and subsequent skin penetration (cutaneous larva migrans; cercarial dermatitis); from direct animal contact (hydatid; toxocariasis) or through insect vectors/intermediate hosts by ingestion (dipylidiasis; *Hymenolepis diminuta* or *Inermicapsifer* infection) or injection by a mosquito (dirofilariasis; *Brugia* infection) [1].

The prevalence of zoonotic helminth infections in man in any region is directly associated with the prevalence of infections in the animal population in that region. A study conducted in Anse-la-Raye, St. Lucia to estimate the prevalence and symptomatology of paediatric toxocariasis recorded 86% seroprevalence of Toxocara canis among the children, though the prevalence of infection in dogs was not abnormally high in the community [5]. Bovine fascioliasis prevalence ranging from 9.2-16.9% was reported from a 5 year study of helminthosis among cattle presented to the Fulani ambulatory clinic in Zaria, Nigeria [6]. A survey of the dog and cat population conducted at New Bussa revealed a significant high frequency of hookworm *Ancylostoma spp*.; *Echinococcus granulosa*, *Dipylidium caninum* in dog; and *Opisthorchis felineus*, *Toxocara cati* and *Capillaria hepatica* in cat [7]. There is a dearth of information on the types of zoonotic helminth infections circulating among humans and animals in Nigeria.

This study is designed to estimate the prevalence of zoonotic helminth infections among animals and human populations in Jos Plateau state; and identify the risk factors for the infection within the population. This might provide a scientific basis for advocacy aimed at unifying human medical and veterinary medical disciplines against zoonotic diseases occurring in the public health arena in line with the "One Health" concept; and also, for the formulation of better control measures in both animals and man.

## Methods

### Study Area

The study was conducted in Jos North and Jos South Local Government Areas (LGA) of Plateau State, Nigeria. The area human population figure was given as 746,016 (FGoN, 2006) approximately a quarter of the population in the entire state. Plateau State has a near temperate climate, though situated in the tropical zone, with an average temperature of between 18 and 22°C. The coldest weather is between December and February. The warmest temperatures usually occur in the dry season months of March and April. The region is endowed with abundant natural resources and livestock. Majority of the people engage in farming as a means of livelihood.

### Human and Animal Data

Laboratory diagnostic records from 2005-2009 were collected from five human health clinics comprising of Government Primary Health Care Centres and private hospitals. Record of helminth investigation and demographic details of the patients including age, sex, address, occupation, educational status and date of test were obtained. Laboratory results with confirmed cases of ZHI were defined as cases for this study while results with non zoonotic helminth were classified as controls. For the animal data, meat inspection records were obtained from the Jos Abattoir and laboratory diagnostic records from a Veterinary Practice, also from 2005 -2009. The record included animal species examined and the species of helminth observed. Zoonotic helminths are categorized as cases and non zoonotic helminthes as controls.

### Statistical Analysis

Statistical analysis was performed using Microsoft Excel^®^ (Redmond, WA, USA) and the Medcalc^®^ 11.1 software (http://www.medcalcsoftware.com/, Belgium). Univariate logistic regression analyses were performed to establish which demographic risk factors were associated with ZHI.

## Results

### Prevalence of Zoonotic Helminth

#### Human

A total of 6689 humans comprising 3304 females and 3885 males were sampled. The patients came from 5 hospitals within the study area. The prevalence ranged from 4.5% to 32.1% within the hospitals. The overall prevalence was found to be 7.8% (95% CI: 7.2-8.5). Zoonotic helminths identified and their prevalences include *Ascaris species* (4.5%); *Taenia species* (1.5%); *Schistosoma species* (1.1%); *Ancylostoma duodenale* (0.6%) and *Strongyloides species* (0.1%) (Table 1).

**Table 1:** Distribution and prevalence of zoonotic helminth infection in humans, Jos Plateau State, 2005-2009

Zoonotic Helminth	No. Positive (N = 6689)	Prevalence (%)	95% CI
*Ancylostoma duodenale*	40	0.59	0.44-0.81
*Ascaris species*	301	4.50	4.03-5.02
*Schistosoma species*	76	1.14	0.91-1.42
*Taeniasis / Cysticercosis*	101	1.51	1.24-1.83
*Strongyloidosis*	6	0.09	0.04-0.19

N = Total Sample Examined; CI = Confidence Interval

#### Animal

A total of 19508 animals comprising 7950 Bovine, 5557 Caprine, 4306 Ovine, 1657 Swine and 38 Canine were sampled. The animals were sampled from an abattoir and a veterinary clinic within the study area. The prevalence in the abattoir was found to be 17.6% and in the clinic 52.2%. The overall prevalence was found to be 18.1% (95% CI: 17.6-18.7). Zonnotic helminths identified and their prevalences include *Fasciola specie*s (12.7%); *Taenia species* (5.0%); *Strongyloides specie*s (0.4%) and *Ascaris specie*s (0.04%) (Table 2).

**Table 2:** Distribution and prevalence of zoonotic helminth infection in animals, Jos Plateau State, 2005-2009

Zoonotic Helminth	No. Positive (N = 19508)	Prevalence (%)	95% CI
*Fasciola species*	2467	12.7	12.2 – 13.2
*Taeniasis / Cysticercosis*	968	5.0	4.7-5.3
*Strongyloidosis*	77	0.4	0.3 – 0.5
*Ascaris species*	8	0.04	0.02 – 0.08

N = Total Sample Examined; CI = Confidence Interval

### Risk Factor for Zoonotic Helminth

#### Human

The positivity of zoonotic helminths according to the demographic characteristics of the human population is given in Table 3. The odds of infection with zoonotic helminth was significantly higher among ages 6-19 (OR 3.2) and 20-60 (OR 2.3) compared to ages 1-5 (*p* < 0.0001). The odds of infection was significantly higher in the peri-urban (OR 1.5; p < 0.0001) and urban (OR 1.3; p = 0.0752) compared to those from rural communities. It is also significantly higher during the rainy season compared to the dry season (OR 1.3; *p* = 0.0328). Among the various occupations, farmers are at significant higher risk. People that attend the private health centres are more prone (OR 1.9; *p* < 0.0001) to be diagnosed with zoonotic helminth compared to those that attend public health facility.

**Table 3:** Risk factor for zoonotic helminth infection in humans, Jos Plateau State, 2005-2009

Variable	*Positive/Total (%)	Odds Ratio	95%CI	p- value
**Age (Year)**				
0-5	35/163 (21.5)	1		
6-19	80/171 (46.8)	3.2	2.0-5.2	< 0.0001
20-60	248/602 (41.2)	2.3	1.7-3.9	< 0.0001
> 60	36/229 (15.7)	0.7	0.4-1.2	0.1464
**Sex**				
Male	190/590 (32.2)	1		
Female	209/576 (36.3)	1.2	0.9-1.5	0.1421
**Locality**				
Rural	273/3731 (7.3)	1		
Peri-Urban	174/1620 (10.7)	1.5	1.3-1.9	< 0.0001
Urban	77/844 (9.1)	1.3	0.9-1.7	0.0752
**Season**				
Dry	285/748 (38.1)	1		
Rainy	239/543 (44.0)	1.3	1.0-1.6	0.0328
**Occupation**				
Business	21/200 (10.5)	0.06	0.02-0.14	< 0.0001
Public servant	16/143 (11.2)	0.06	0.02-0.16	< 0.0001
Student	63/314 (20.1)	0.12	0.05-0.29	< 0.0001
Housewife	34/169 (20.1)	0.12	0.05-0.30	< 0.0001
Farmer	17/25 (68.0)	1		
**Education**				
No formal Education	19/196 (9.7)	1.5	0.8-2.6	0.1876
Educated	42/615 (6.8)	1		
**Health facility**				
Public	194/1046 (18.6)	1		
Private	330/1097 (30.1)	1.9	1.5-2.3	< 0.0001
**Diagnostic facility**				
Vet Clinic	119/230 (51.7)	5	3.9-6.5	<0.0005
Abattoir	3401/19294 (17.6)	1		

* Number positive for Zoonotic Helminth / Total Sample Examined, CI = Confidence Interval

#### Animals

The odds of infection with zoonotic helminth was higher among bovine (OR 1.4) compared to canine but with no statistical significance (*p* = 0.3875) but lower among caprine (OR 0.6), ovine (OR 0.7) and swine (OR 0.7). The odds of finding zoonotic helminth are significantly higher in the clinic compared to the abattoir (OR 5.0; *p* < 0.0005).

## Discussion

Our study indicated that the mean prevalence of zoonotic helminth among humans sampled in the Jos North and Jos South LGA of Plateau state was 7.8%. This was the first such study in this part of the state (to the best of our knowledge). This prevalence is found to be high; however, considering the nature of the disease, infection represents a significant health threat to humans. The prevalence of Ascaris, hookworm (Ancylostoma), Strongyloides was however found to be lower than those reported from a survey conducted among inhabitant of Tan-nan village, Nantou, Taiwan [8].

Our study found a higher risk among age groups 6-19 years (46.8%) and 20-60 years (41.2%) compared to age 0-5 years (21.5%). This might be due to the fact that activities and exposure to risk factors is higher among this age group. This value obtained among age 0-5 years is similar to the findings from a study conducted among children in the state of Minas Gerais where 26.9% prevalence was reported among ages 0-5 years and 21.2% in 5-10 years [9]. Also similar to the report of a study conducted among school children from Ota, Ogun state Nigeria [10]. The risk of infection was higher among females (OR: 1.2), different from higher risk in male (OR: 2.7) reported by [9]. This might be due to the fact that female engages in farming activities more than their male counterpart in our study area.

The risk of infection was found to be significantly higher in the sub-urban (OR: 1.5) and urban (OR: 1.3) areas compared to the rural areas. This might be due to the fact that urban and sub-urban dwellers seek medical attention more compared to rural dwellers in our study area. This finding is contrary to the finding from a study conducted in Santa Cruz, Bolivia in 2000 [11]. Increased risk recorded during the rainy season (OR: 1.3) in our study might be due to the persistent erosion which washes helminth oocysts from contaminated soil into surface water and streams which serves as sources of drinking water for most communities in developing countries.

The risk is significantly higher among farmers compared to other occupation examined probably due to the fact that farmer are more exposed to the sources of infection which include exposure to animals and soil which serve as reservoir of infection [5]. The odds of diagnosing zoonotic helminth is 2 times higher in the private compared to public health facility as found by our study. Private health facility are observed to be well equipped to carry out laboratory diagnosis and open to patient all year round unlike public health facility that are sometimes closed due to industrial action.

Among animals, our study indicated that the mean prevalence of zoonotic helminth in the Jos North and Jos South LGA of Plateau state was 18.1%. The prevalence was higher in the animal hospital (52.2%) compared to the abattoir (17.6%). The hospitals receive and attend more to sick animals and diagnosis is supported by laboratory screening hence records more cases compare to abattoir where diagnosis is mostly based on gross lesions. The risk of infection was higher in bovine (OR: 1.4) compared to canine. This might be due to the fact that canine are less exposed to contamination than bovine.

## Conclusion

A high prevalence of infection among animals poses a great threat to infection in human and public health. There is a higher risk of infection among humans age 6-60 years, peri-urban dwellers and farmers. Uncontrolled incidence in these species will result in perpetual contamination of the environment and increased burden of infection in both animal and human.
